# Dietary supplementation with *L*-citrulline improves amino acid composition and broiler performance, and modulates gut microbiota

**DOI:** 10.3389/fmicb.2025.1551012

**Published:** 2025-02-13

**Authors:** Yan Ma, Tingting Liu, Shuaihu Chen, Hong Shen, Jungang Wang

**Affiliations:** ^1^Laboratory of Animal Nutrition and Feed Science, College of Animal Science and Technology, Shihezi University, Shihezi, China; ^2^College of Agriculture, Shihezi University, Shihezi, China

**Keywords:** *L*-citrulline, broilers, production performance, amino acid composition, intestinal health

## Abstract

Poultry lacks carbamoyl phosphate synthetase, which is a key enzyme in the endogenous synthesis of arginine, thus poultry must obtain arginine from their diet. Citrulline (*L*-Cit), as a precursor of arginine, produces the same biological effects as arginine, and may even be more effective. In these experiments, we discovered that the addition of *L*-Cit significantly increased production performance, antioxidant and immune properties, and modulated the intestinal microbiota. The study involved 240 1-day-old male yellow-feathered broilers randomly assigned to one of four treatment groups: control (CON), 0.5% *L*-Cit, 1% *L*-Cit, and 1.5% *L*-Cit, with 10 replicates per group and six birds per replicate. The feeding trial lasted for 63 days. The body weight at 42 days and 63 days, as well as the average daily weight gain from day 1 to 63, increased linearly and quadratically with the addition of *L*-citrulline. The plasma concentrations of citrulline, ornithine, and arginine increased linearly with the dose of *L*-Cit. *L*-citrulline increased total antioxidant capacity (T-AOC) and superoxide dismutase (SOD) levels and decreased interleukin-1β (IL-1β), IL-6, and tumor necrosis factor alpha (TNF-*α*). Dietary addition of 1% *L*-Cit also significantly altered the overall composition of the broiler intestinal microbiota, increasing the relative abundance of Prevotellaceae_UCG-001 while reducing the relative abundance of Synergistota and Campylobacterota. This indicates that *L*-Cit can enhance the production performance of broilers, and improve antioxidant activity and immune functions, thereby protecting intestinal health. The optimum dietary amount of *L*-Cit is 1 to 1.5%.

## 1 Introduction

In the context of a comprehensive ban on the use of antibiotic growth promoters, adopting nutritional strategies to prevent intestinal damage and promote healthy growth in animals has become a research hotspot (Mahasneh et al., 2024). Research has proven that certain amino acids can protect the intestinal barrier (Wang et al., 2015). Among them, arginine, as a multifunctional basic amino acid, plays a wide range of physiological roles in the animal body. Arginine in animals is mainly derived from the diet and from proteolysis. However, the metabolic pathways of poultry differ from those of other animals, particularly with regard to ammonia metabolism. Chickens are unable to convert ammonia to urea through the ornithine cycle as mammals do due to the lack of carbamoylphosphate synthetase, an enzyme that plays a key role in the urea cycle, catalyzing the irreversible synthesis of carbamoylphosphate from NH_3_, CO_2_, and 2 molecules of ATP. However, in poultry, the absence of this enzyme requires the intake of arginine from diet to process ammonia in the body and maintain normal metabolism (Liu and Kim, 2023).

*L*-citrulline (*L*-Cit), also known as carbamoylornithine, is a non-protein *α*-amino acid, which is mainly synthesized by intestinal cells under the action of ornithine carbamoyltransferase on glutamine, and is transported in the blood to the kidneys, where it generates arginine under the catalytic action of arginine succinate synthetase and arginine succinate cleavage enzyme (Moinard and Cynober, 2007). The arginine synthesized from *L*-Cit is used as a precursor by nitric oxide synthase to generate nitric oxide (NO), which can activate the mitogen-activated protein kinase (MAPK) signaling pathway. This in turn upregulates the expression of the transcription factors, nuclear factor-κB (NF-κB) and activator protein-1 (AP-1), which are necessary for T cell activation, differentiation and proliferation (Lan et al., 2020). It can also alleviate oxidative stress in the body by activating antioxidant enzymes (Van De Poll et al., 2007). Administering *L*-Cit orally at a dose of 145 mg/kg twice daily to suckling piglets results in higher plasma concentrations of arginine, indicating that the small intestine of suckling piglets has a significant capacity to synthesize arginine from *L*-Cit (Wu, 1995). Supplementing 7-day-old newborn piglets with proline, ornithine, citrulline, and arginine for 5 days showed that piglets fed diets containing *L*-Cit and arginine had higher plasma concentrations and flows of arginine, with a lower conversion efficiency of proline to arginine (Urschel et al., 2006). Adding 12 g/d of *L*-Cit to the diet of rams improved the composition of gut microbiota, thereby enhancing meat quality, improving freeze resistance, and promoting the development of spermatogenic cells in the testes (Fan et al., 2024). The above research findings support the hypothesis that *L*-Cit added to the diet can be efficiently converted into arginine within the body, thereby meeting the body’s requirements for arginine and its biological effects in promoting healthy growth.

Currently, in poultry breeding, amino acid deficiency or insufficient protein deposition can impair mucosal integrity, microbiota balance, and immune function of the intestine, which can lead to a decline in production performance. *L*-Cit is a non-essential amino acid that has rarely been studied as a supplement in poultry nutrition. *L*-Cit can be produced at a low cost and has a price advantage compared to arginine. Therefore, this study was conducted to investigate the effects of adding different amounts of *L*-Cit under the same feeding conditions on plasma amino acid concentrations, antioxidant and immune properties, and intestinal health of broilers, aiming to provide a scientific basis for its application in poultry production.

## 2 Materials and methods

### 2.1 Ethical statement

The study was approved by the Animal Experimentation Ethics Committee of the School of Animal Science and Technology, Shihezi University. Broilers were housed and handled according to committee guidelines and euthanized humanely. All actions were governed by the principles of minimizing animal suffering (Approval #2023–031).

### 2.2 Broiler experimental design, animal feeding

For this investigation, we selected 240 healthy one-day-old male broiler chickens of similar weight, and organized them into four treatment groups based on their weight: control (CON), 0.5% *L*-Cit, 1% *L*-Cit, and 1.5% *L*-Cit. Each group had six replicates, with 14 chickens per replicate. After rearing on the ground until 7 days of age, the chickens were housed in three-tiered cages, with each replicate randomly distributed among the cages. Feeding began on the second day of age, using a corn-soybean meal-based diet formulated according to the standards for broilers set by the United States National Research Council (NRC), with the feed ingredients and nutritional levels listed in Table 1. All tests were performed in the same facility, where the temperature was thermostatically controlled at 32–35°C on the first day, then gradually lowered to 22°C and maintained there for the remaining 2 weeks. The broilers were given food and water *ad libitum*. Animals were immunized according to schedule and cages were disinfected following farm procedures. The experimental duration was 63 days. *L*-Cit was purchased from Shandong Pingju Biotechnology Co., Ltd. It appears as a white powder, with an active ingredient content of 99.1% and an ash content of 0.05%.

**Table 1:** Feed ingredients and nutrient concentrations at different ages.

Item	1–21 days old	22–42 days old	43–63 days old
Ingredients			
Corn	62	64	70
Soybean meal	23	21	15
Cottonseed meal	6	6	6
Wheat bran	3.47	3.47	3.47
CaHPO_4_	1.24	1.24	1.24
Limestone	1.3	1.3	1.3
Salt	0.24	0.24	0.24
Lys	1.33	1.33	1.33
Met	0.25	0.25	0.25
Thr	0.17	0.17	0.17
Premix	1	1	1
Nutrient levels			
ME(MJ/kg)	13.50	13.00	12.50
DM	90.01	90.64	91.92
Organics	94.25	93.73	95.67
CP	22.26	19.70	18.14
EE	3.13	3.67	4.33
ADF	4.74	4.81	4.71
NDF	11.49	11.41	11.78
P	0.57	0.57	0.59
Ca	1.24	1.49	1.27
Lys	1.12	0.85	0.76
Met	0.49	0.45	0.43
Thr	0.80	0.64	0.59

Per kg of feed, the premix provides:VitA 180,000 IU; VitD 70,000 IU; VitE 450 IU; VitK 30 mg; VitB 70 mg; niacin 600 mg; calcium pantothenate 260 mg; biotin 1.7 mg; folic acid 17 mg; Fe 10,000 mg; Cu 350 mg; Mn 1,500 mg; Zn 2000 mg; Ca 14 mg; P 6 mg; sodium chloride 7 mg; and methionine 3 mg.

### 2.3 Sample collection

The body weight (BW) and feed consumption in each group were recorded weekly, and the average daily feed intake (ADFI) and average daily gain (ADG) were also calculated. At 63 days of age (doa), six broilers were randomly selected from each group for venous blood collection. After clotting, the serum was collected by centrifugation at 3000 g for 10 min at 4°C. Birds were then euthanized and samples of chest muscle, liver, spleen, and thymus were removed. The contents of the cecum was squeezed out into a 2 mL lyophilizer tube. The ileal and jejunal mucosa were scraped into a 2 mL lyophilizer tube, flash frozen in liquid nitrogen, and stored (−80°C) for later analysis.

### 2.4 Growth and carcass traits

BW and feed intake (FI) of broilers were measured on days 1, 21, 42 and 63 of the trial and F/G, ADG, and ADFI were calculated. At the end of the experimental period, one broiler near the average BW was euthanized after 12 h of fasting and carcass weight, live weight, eviscerated weight, and abdominal fat weight were determined to calculate the carcass yield, eviscerated yield, half eviscerated yield, breast meat yield, thigh meat yield and abdominal fat yield.

$$\mathrm{Carcass yield}=\left(\mathrm{carcass weight}/\mathrm{live weight}\right)\times 100\%$$

$$\mathrm{Eviscerated yield}=\left(\mathrm{eviscerated weight}/\mathrm{live weight}\right)\times 100\%$$

$$\mathrm{Half eviscerated yield}=\left(\begin{array}{l} \mathrm{half eviscerated weight}/\\ \mathrm{live weight} \end{array}\right)\times 100\%$$

$$\mathrm{Breastyield}=\left(\mathrm{breast muscle weight}/\mathrm{eviscerated weight}\right)\times 100\%$$

$$\mathrm{Thighyield}=\left(\mathrm{thigh muscle weight}/\mathrm{eviscerated weight}\right)\times 100\%$$

$$\begin{array}{l} \mathrm{Abdominal}\;\mathrm{fat}\;\mathrm{yield}=\mathrm{abdominal}\;\mathrm{fat}\;\mathrm{weight}\\ /\left(\mathrm{eviscerated weight}+\mathrm{abdominal}\;\mathrm{fat}\;\mathrm{weight}\right)\times 100\% \end{array}$$

### 2.5 Meat quality

The pH of the LD and BF muscle was measured at 0.75 h and 24 h using a Magnetics PHBJ-260 pH meter (INESA Scientific Instrument Co., Ltd., Shang-hai, China). The pH of the breast meat was measured according to the method of Eltazi et al. (2014) with modifications. Meat color was established by colorimeter, calibrated with black and white plates before measuring the meat samples.

Muscle samples were heated at 80°C to an internal temperature of 70°C, and the cooking loss was expressed as the percent reduction in weight after heating. A C-LM3B texture analyzer (Beijing Tianxiang Feiyu Instrument Co., Ltd., Nanjing, China) was used to measure the Warner-Bratzler shear force (expressed in kgf) from six 1.27 cm diameter columnar muscle samples parallel to muscle fiber orientation.

For the determination of drip loss, the left pectoral muscle was taken and cut into 2 cm x 3 cm x 5 cm pieces. The meat pieces were attached to hangers and initial weight, W1, was determined. The pieces were hung in a refrigerator at 4°C for 24 h, then re-weighed (W2) and drip loss was calculated according to the formula: drip loss rate = [(W1-W2) /W1] × 100%.

The moisture and crude protein and fat in the meat were determined by the AOAC method (AOAC International, 2000). Samples were weighed then dried in an oven at 105°C to constant weight to measure water content. Crude protein was quantitated by Kjeldahl nitrogen assay, and crude fat by Soxhlet extraction.

### 2.6 Amino acid content

For determination of free amino acid content, 50 μL of plasma was mixed with 50 μL of protein precipitator (including NVL), vortexed, centrifuged at 4°C for 4 min at 13,200 g to deproteinize; then, 8 μL of supernatant was added to 42 μL labeled buffer and 20 μL derivative reagent and incubated at 55°C for 15 min. Derivatized samples were cooled, centrifuged, and quantitatively analyzed by HPLC tandem mass spectrometry.

### 2.7 Plasma biochemistry, antioxidant and immune indexes

Plasma IgA, IgG, IgM, IL-1β, IL-6, and tumor necrosis factor TNF-*α* content, malondialdehyde (MDA) concentration, superoxide dismutase (SOD) activity, and total antioxidant capacity (T-AOC) were measured by kits (Beijing Huaying Biotechnology Institute). The plasma levels of alanine aminotransferase (ALT), total protein (TP), albumin (ALB), globulin (GLB), aspartate aminotransferase (AST), urea (UREA), glucose (GLU), triglycerides (TG) and total cholesterol (TC) were determined with an AU480 automatic biochemical analyzer.

After blood collection, each animal was weighed and euthanized by cervical dislocation. The thymus, spleen, and bursa of Fabricius were removed, weighed, and the weights were expressed as percentage of total BW: percent relative organ weight = (organ weight/body weight) × 100.

### 2.8 Bacterial DNA purification and 16S rRNA gene sequencing

DNA was purified from cecal contents by the cetyltrimethylammonium bromide (CTAB) method and used as template for PCR amplification with specific primers. The PCR products were quantified by fluorometry (Qubit 3.0), and sequenced on an Illumina PE300 platform at the Beijing NOhe Zhiyuan Technology Co.

The raw data from 16S rRNA gene sequencing was QC processed with QIIME2, to remove low-quality reads and potential contaminants. The SILVA database was selected for alignment, and a 97% sequence similarity cut-off was used to identify operational taxonomic units (OTUs). Alpha diversity of the microbial community was assessed by Chao1, ACE and Shannon indexes in QIIME2. Beta diversity was determined by principal coordinates analysis (PCoA). Differences in relative bacterial abundance were evaluated by the nonparametric Kruskal-Wallis rank-sum test, and the difference between groups was analyzed by Student’s *t*- test. Bacterial markers differentiating the various microbial communities were determined by linear discriminant analysis (LDA) effect size (LEfSe) (LDA > 4, *p* < 0.05). Pearson’s correlation coefficients (*p* < 0.05) were used to highlight the co-occurrence of microbial communities among the top 35 genera.

### 2.9 Extraction of RNA and quantitative real-time PCR analysis

A TRIzol kit (TransGen, Beijing, China) was employed to extract total RNA and its purity and concentration were determined spectrophotometrically (Thermo Fisher Scientific, Waltham, MA, United States). A reverse transcription kit was used to convert total RNA into cDNA, which was analyzed in a 20 μL reaction containing 15 μL of PerfectStart Green qPCR SuperMix, 10 ng of cDNA, and 0.2 μM forward and reverse primers on a Roche LightCycler 96. Thermocycling conditions: initial denaturation at 95°C for 30s, followed by 50 cycles of denaturation at 95°C for 10 s, annealing at 60°C for 15 s, elongation at 72°C for 10s. Samples were run in triplicate, and relative expression was determined using the 2^−∆∆Ct^ method. Primers for the broiler genes, *TNF-α, IL-1β, IL-6, Occludin, Claudin-1,* and *ZO-1* were used, with β-actin serving as the reference gene (Table 2).

**Table 2:** Primer sequences of target genes and the reference gene, β-actin.

Gene name	Primer sequence (5′-3′)
TNF-α	F: GGCAATGAACCCTCCCCAGTA
	R: GGTTACAGGAAGGGCAACTCATC
IL-1*β*	F: CAGCCTCAGCGAAGAGACCTT
	R: ACTGTGGTGTGCTCAGAATCC
IL-6	F: CGCCTTTCAGACCTACCT
	R: GGATTGTGCCCGAACTAA
Occludin	F: ACGGCAGCACCTACCTCAA
	R: GGGCGAAGAAGGAGATGAG
Claudin-1	F: TTCATGATGCCTGCTCTTGTG
	R: CCTGAGCCTTGGTACATTCTTGT
ZO-1	F: GGGATGTTTATTTGGGCGGC
	R: TCACCGTGTGTTGTTCCCAT
β-Actin	F: GAGAAATTGTGCGTGACATCA
	R: CCTGAACCTCTCATTGCCA

### 2.10 Statistical analyses

The experimental data were initially sorted by Excel 2010, and the ANOVA program of SPSS 26.0 was used for one-way ANOVA analysis. If the differences were significant, Duncan’s method was used for multiple comparison, and orthogonal polynomial comparison was used to test the linear and quadratic effects of changes in relevant indicators. The test results were expressed as mean and standard error (SE); *p* < 0.05 was the significant difference level, and *p* < 0.01 was the extremely significant difference level.

## 3 Results

### 3.1 Growth performance

Table 3 shows that the BW at 42 doa was increased linearly (*p* = 0.009) and quadratically (*p* = 0.043) by the addition of *L*-Cit, with the 0.5, 1, and 1.5% groups being significantly higher than the control (*p* < 0.05). At 63 doa, BW in the *L*-Cit groups had increased linearly (*p* = 0.006) with all experimental groups being significantly higher than the CON group (*p* < 0.05). The average daily weight gain of yellow-feathered broilers from day1 to day63 also showed a linear increase (*p =* 0.007) with *L*-Cit supplementation.

**Table 3:** Effect of dietary level of L-Cit on growth performance of broilers.

Items	Groups				SE	*p-* value		
	CON	0.5% *L*-Cit	1.0% *L*-Cit	1.5% *L*-Cit		Total	Linear	Twice
1–21 days of age								
BW(g) at 1 day of age	32.28	32.22	32.93	32.50	1.241	0.239	0.104	0.226
BW(g) at 21 days of age	446.66	443.33	460.00	438.46	7.738	0.835	0.908	0.580
ADFI	31.75	32.72	33.28	32.87	0.328	0.419	0.195	0.302
ADG	18.65	20.77	20.10	20.04	0.528	0.576	0.478	0.326
F/G	1.70	1.58	1.67	1.68	0.059	0.715	0.727	0.385
22–42 days of age								
BW(g) at 42 days of age	1377.27^b^	1505.00^a^	1578.75^a^	1527.50^a^	27.473	0.010	0.009	0.043
ADFI	91.08	90.61	91.26	90.49	0.148	0.194	0.388	0.603
ADG	42.69	47.54	52.07	51.51	1.669	0.166	0.041	0.404
F/G	2.23	1.93	1.76	1.82	0.078	0.142	0.046	0.224
43–63 days of age								
BW(g) at 63 days of age	2467.83^b^	2819.00^a^	2776.67^a^	2864.00^a^	50.286	0.010	0.006	0.121
ADFI	130.77	131.23	131.73	132.12	1.291	0.987	0.720	0.989
ADG	53.31	63.29	62.78	66.59	2.375	0.233	0.070	0.510
F/G	2.58	2.11	2.19	2.01	0.104	0.219	0.082	0.467
1–63 days of age								
ADFI	73.86	72.67	73.28	76.53	2.391	0.952	0.709	0.666
ADG	38.75^b^	44.23^a^	43.55^a^	44.94^a^	0.792	0.012	0.007	0.128
F/G	1.92	1.64	1.65	1.66	0.056	0.226	0.123	0.197

Values followed by different lower-case letters are significantly different (at *p* = 0.05), values followed by different upper-case letters are significantly different (at *p* < 0.01), and there is no significant (*p* > 0.05) difference with or without the same letter.

### 3.2 Carcass traits

Table 4 shows that the live weight before slaughter was increased linearly (*p* = 0.043) and quadratically (*p* = 0.017) by the addition of *L*-Cit, and all experimental animals were significantly heavier than control. *L*-Cit also increased the carcass weight significantly, both linearly (*p* = 0.028) and quadratically (*p* = 0.030) in all experimental groups relative to the control. *L*-Cit also increased the total net weight linearly (*p* = 0.020), where the 1.5% group was significantly higher than the CON group, showing an increase of 18.92% (*p* < 0.05). The weight of the leg muscles increased linearly (*p* = 0.011) and quadratically (*p* = 0.010) with the addition of *L*-Cit.

**Table 4:** Effect of dietary level of L-Cit on broiler carcasses.

Items	Groups				SE	*p-* value		
	CON	0.5% *L*-Cit	1.0% *L*-Cit	1.5% *L*-Cit		Total	Linear	Twice
Live weight, g	2434.50^b^	2735.00^a^	2746.67^a^	2911.67^a^	49.380	0.030	0.043	0.017
Dressed carcass weight, g	2313.17^b^	2605.00^a^	2621.50^a^	2765.00^a^	47.990	0.039	0.028	0.030
Carcass yield, %	95.05	95.17	95.47	94.96	0.256	0.226	0.104	0.279
Half-eviscerated weight, g	2185.00	2450.00	2459.33	2213.50	84.220	0.719	0.618	0.690
Half-eviscerated yield, %	89.81	89.55	89.53	76.57	2.718	0.406	0.654	0.426
Eviscerated weight, g	1810.00^b^	2017.67^ab^	2028.00^ab^	2152.50^a^	36.268	0.041	0.020	0.050
Eviscerated yield, %	74.41	73.79	73.80	73.93	0.266	0.084	0.114	0.021
Breast muscle weight, g	156.55	154.28	165.78	183.83	4.321	0.210	0.090	0.549
Breast, %	8.64	7.70	8.16	8.54	0.191	0.585	0.885	0.410
Thigh muscle weight, g	172.80^b^	205.43^a^	214.11^a^	231.18^a^	5.584	0.010	0.011	0.010
Thigh, %	9.51	10.19	10.55	10.73	0.153	0.101	0.088	0.030
Abdominal fat weight, g	53.72	71.60	62.63	66.97	4.505	0.312	0.439	0.074
Abdominal fat, %	2.86	3.36	2.95	2.97	0.183	0.295	0.145	0.138

Values followed by different lower-case letters are significantly different (at *p* = 0.05), values followed by different upper-case letters are significantly different (at *p* < 0.01), and there is no significant (*p* > 0.05) difference with or without the same letter.

### 3.3 Meat quality

*L*-Cit increased the post-mortem pH at 24 h both linearly (*p* = 0.026) and quadratically (*p* = 0.001), and the pH at 24 h post-mortem in the 0.5 and 1% groups was significantly higher than that of the CON group (*p* < 0.01), while the pH in the 1.5% group was significantly higher than that of the control (*p* < 0.05; Table 5).

**Table 5:** Effect of dietary level of L-Cit on meat quality of broilers.

Items	Groups				SE	*p-* value		
	CON	0.5% *L*-Cit	1.0% *L*-Cit	1.5% *L*-Cit		Total	Linear	Twice
Shear force/N	53.24	47.51	45.76	51.47	2.290	0.864	0.906	0.417
Breast muscle color 1 h after slaughter	82.30	79.04	81.36	82.16	0.849	0.776	0.657	0.517
Breast muscle color 24 h after slaughter	70.90	67.54	71.615	73.55	1.255	0.740	0.580	0.888
pH 1 h after slaughter	5.30	5.25	5.34	5.25	0.037	0.791	0.439	0.587
pH 24 h after slaughter	5.00^bB^	5.22^aA^	5.22^aA^	5.14^aB^	0.026	0.002	0.026	0.001
Drip loss, %	7.68	8.02	5.58	6.76	0.612	0.465	0.135	0.917
Protein, %	20.83	20.74	20.74	20.57	0.041	0.271	0.044	0.663
Moisture, %	70.62	70.40	69.85	69.55	0.180	0.336	0.275	0.124
Fat, %	5.90	7.13	6.98	7.36	0.226	0.166	0.035	0.330

Values followed by different lower-case letters are significantly different (at *p* = 0.05), values followed by different upper-case letters are significantly different (at *p* < 0.01), and there is no significant (*p* > 0.05) difference with or without the same letter.

### 3.4 Amino acid concentration and related metabolite content

The addition of *L*-Cit resulted in a linear increase in citrulline (*p =* 0.020), ornithine (*p* = 0.003), and arginine (*p* < 0.001), and the concentration of citrulline in the experimental groups was significantly higher than in the control (Table 6). For alkaline amino acids, the histidine content showed a linear increase with the addition of *L*-Cit (*p* = 0.012). Regarding neutral amino acids, the tryptophan content demonstrated a linear increase with *L*-Cit supplementation (*p* < 0.001) and a trend of first increasing and then decreasing (*p* = 0.004). In terms of related metabolites, the NO content exhibited a linear increase (*p* < 0.001) and a quadratic increase (*p* = 0.014), while urea levels increased linearly with *L*-Cit addition (*p* < 0.001) and showed a trend of first increasing and then decreasing (*p* = 0.024). The content of non-essential amino acids increased linearly with the addition of *L*-Cit (*p* = 0.016), whereas the ratio of essential to non-essential amino acids decreased linearly with *L-*Cit addition (*p* = 0.001).

**Table 6:** Effect of dietary level of L-Cit on concentration of plasma amino acids and related metabolites in broilers (μg/mL).

Items	Groups				SE	*p-* value		
	CON	0.5% *L*-Cit	1.0% *L*-Cit	1.5% *L*-Cit		Total	Linear	Twice
Citrulline	0.81^b^	7.62^ab^	10.55^a^	13.98^a^	1.601	0.013	0.020	0.510
Ornithine	4.02^b^	6.22^ab^	8.26^a^	9.53^a^	0.729	0.025	0.003	0.701
Arginine	60.90^bB^	89.99^aAB^	106.37^aA^	112.08^aA^	5.984	0.002	<0.001	0.192
Basic amino acids								
Histidine	15.66^b^	19.22^ab^	21.66^a^	20.96^a^	0.854	0.044	0.012	0.163
Lysine	69.35	63.53	46.11	47.98	8.575	0.177	0.046	0.654
Acidic amino acids								
Aspartic acid	10.34	12.77	14.98	10.68	1.323	0.105	0.608	0.027
Glutamic acid	33.25	39.19	36.40	37.43	4.572	0.704	0.556	0.507
Neutral amino acids								
Glycine	37.02	38.17	42.05	39.91	1.333	0.601	0.322	0.558
Alanine	60.72	59.60	62.99	57.89	6.796	0.911	0.825	0.701
Serine	45.48	39.37	46.79	45.69	1.556	0.341	0.565	0.424
Proline	29.01	37.12	35.67	33.39	1.385	0.178	0.330	0.063
Valine	26.24	23.10	23.85	21.79	0.804	0.266	0.088	0.733
Threonine	64.14	59.23	54.51	59.85	3.185	0.795	0.567	0.458
Cysteine	13.82	14.72	13.87	9.92	0.466	0.172	0.091	0.140
Isoleucine	13.56	11.98	12.31	11.85	0.467	0.588	0.279	0.570
Asparagine	3.58	5.76	5.21	6.18	1.966	0.587	0.264	0.671
Glutamine	104.49	128.69	124.23	113.99	8.063	0.227	0.538	0.061
Methionine	9.71	12.56	11.09	10.37	0.957	0.265	0.913	0.098
Phenylalanine	14.61	16.12	16.28	15.29	0.489	0.628	0.632	0.233
Tryptophan	11.51^cC^	15.27^bBC^	21.39^aA^	18.02^bAB^	0.967	<0.001	<0.001	0.004
Tyrosine	28.88	29.17	26.78	22.61	1.932	0.210	0.059	0.353
Leucine	24.02	28.16	28.42	25.92	1.057	0.439	0.539	0.137
Total amino acids	975.41	1060.70	1072.85	1082.96	29.874	0.600	0.348	0.345
Essential amino acids	233.13	229.96	213.97	221.06	8.014	0.728	0.290	0.994
Non-essential amino acids	509.15^b^	612.92^ab^	655.03^a^	638.58^a^	20.966	0.046	0.016	0.114
EAA/NEAA	0.46^aA^	0.38^bA^	0.33^bB^	0.33^bB^	0.016	0.004	0.001	0.085
Related metabolites								
NO, μmol/L	33.99^cC^	36.75^cBC^	40.49^bB^	48.69^aA^	1.246	<0.001	<0.001	0.014
UREA, mmol/L	0.23^cB^	0.33^bAB^	0.47^aA^	0.40^abA^	0.118	0.001	<0.001	0.024
AN, μmol/L	18.48	22.81	22.38	23.13	0.781	0.115	0.05	0.232

### 3.5 Plasma biochemical indices

Table 7 shows that there were no significant differences in AST, TP, ALB, GLB, AST, GLU, TG, or TC among all groups (*p* > 0.05).

**Table 7:** Effect of dietary level of L-Cit on plasma indexes of broilers.

Items	Groups				SE	*p-* value		
	CON	0.5% *L*-Cit	1.0% *L*-Cit	1.5% *L*-Cit		Total	Linear	Twice
ALT,U/L	10.30	7.77	7.90	6.60	0.595	0.162	0.041	0.585
TP, g/L	39.07	40.57	45.95	36.1	1.497	0.120	0.781	0.055
ALB, g/L	16.85	17.32	19.38	15.77	0.573	0.152	0.809	0.074
GLB, g/L	22.22	23.25	26.57	20.33	1.077	0.224	0.804	0.095
AST, U/L	49.21	38.51	32.14	23.28	7.257	0.749	0.533	0.835
GLU, mmol/L	12.79	16.19	17.02	15.54	0.665	0.122	0.118	0.063
TG, mmol/L	0.54	0.79	1.30	0.53	0.134	0.135	0.671	0.057
TC, mmol/L	3.97	3.74	4.10	3.39	0.144	0.331	0.288	0.407

### 3.6 Antioxidant and immune indexes

Table 8 shows that the liver index increased linearly with the addition of *L*-Cit (*p* = 0.021), and the liver index in each experimental group was significantly higher than in control (*p* < 0.05).

**Table 8:** Effect of dietary level of L-Cit on meat quality of broilers.

Items	Groups				SE	*p-* value		
	CON	0.5% *L*-Cit	1.0% *L*-Cit	1.5% *L*-Cit		Total	Linear	Twice
Thymic index, %	0.14	0.17	0.18	0.19	0.013	0.859	0.325	0.611
Bursa index, %	0.17	0.14	0.16	0.14	0.009	0.523	0.138	0.966
Spleen index, %	0.14	0.13	0.16	0.13	0.005	0.374	0.411	0.787
Liver index, %	1.74^b^	2.05^a^	2.05^a^	2.07^a^	0.050	0.046	0.021	0.123

Values followed by different lower-case letters are significantly different (at *p* = 0.05), values followed by different upper-case letters are significantly different (at *p* < 0.01), and there is no significant (*p* > 0.05) difference with or without the same letter.

Table 9 shows that the T-AOC and the SOD activity increased linearly with the amount of *L*-Cit added (*p* < 0.001). The T-AOC in the 1.5% group was significantly greater than in the CON group (*p* < 0.001), and the SOD in the 1.5% group was significantly higher than in other groups. Conversely, the concentration of MDA, IL-1β, IL-6, and TNF-*α* decreased linearly with increasing addition of *L*-Cit (*p* < 0.001). The amount of MDA in the 1 and 1.5% groups was significantly lower than in control (*p* < 0.001), and the level of IL-1β, IL-6, and TNF-α in each experimental group was significantly lower (*p* < 0.001).

**Table 9:** Effect of dietary level of L-Cit on antioxidant and immune indexes of broilers.

Items	Group				SE	*p-* value		
	CON	0.5% *L*-Cit	1.0% *L*-Cit	1.5% *L*-Cit		Total	Linear	Twice
T-AOC, U/mL	8.66^cB^	10.37^bAB^	11.09^abA^	12.47^aA^	0.372	<0.001	<0.001	0.739
SOD, U/mL	59.99^cC^	73.39^bB^	77.07^bB^	95.28^aA^	2.851	<0.001	<0.001	0.322
MDA, nmol/mL	3.39^aA^	2.78^bA^	1.77^cB^	1.44^cB^	0.187	<0.001	<0.001	0.494
IgA, g/L	2.36	2.09	3.35	1.77	0.350	0.447	0.866	0.363
IgG, g/L	4.32	3.90	7.00	4.47	1.725	0.778	0.735	0.655
IgM, g/L	1.7676	1.5736	1.818	1.357	0.093	0.663	0.432	0.633
IL-1β, pg./mL	24.79^aA^	19.42^bAB^	16.63^bB^	15.38^bB^	0.524	0.001	<0.001	0.170
IL-6, pg./mL	148.04a^A^	116.70b^B^	101.94b^B^	90.05^cB^	5.780	<0.001	<0.001	0.148
TNF-α, pg./mL	54.16^aA^	49.64^bAB^	44.63^cBC^	38.49^dC^	1.101	<0.001	<0.001	0.595

Values followed by different lower-case letters are significantly different (at *p* = 0.05), values followed by different upper-case letters are significantly different (at *p* < 0.01), and there is no significant (*p* > 0.05) difference with or without the same letter.

### 3.7 Microbial composition

**Figure 1:**
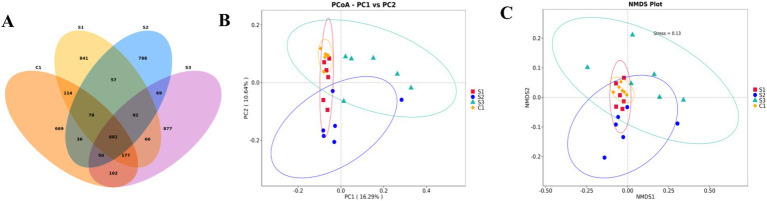
The broilers exhibit rich gut microbiota diversity. **(A)** Venn diagram based on OTUs. **(B)** Results based on PCoA, weighted principal coordinate analysis. **(C)** Results based on NMDS, weighted principal coordinate analysis. (S3, 1.5% group; S2, 1% group; S1, 0.5% group; C1, control group).

After OTU analysis, Venn diagrams for each sample were constructed showing the different groups (Figure 1A). There are 681 OTUs common to the four groups, with the CON group having 669 unique OTUs, the 0.5% group 841, the 1% group 788, and the 1.5% group 877. The Shannon and Simpson indexes of the 1% group were significantly lower than those of the control and other experimental groups, while the Pielou Evenness index was significantly reduced compared with the other experimental groups (*p* < 0.05; Table 10). The dominance index of the 1% group was significantly greater than that of the control and the other experimental groups (*p* < 0.05). Based on PCoA (Figure 1B) and NMDS (Figure 1C) analyses, there is a clear separation between the CON group and the 1% group, indicating a significant difference in gut microbiota between the CON group and the 1% group.

**Table 10:** Effect of dietary level of L-Cit on microbial diversity in cecum of broilers.

Items	Group				SE	*p-* value		
	CON	0.5% *L*-Cit	1.0% *L*-Cit	1.5% *L*-Cit		Total	Linear	Twice
Chao1	773.49	773.31	618.06	762.35	23.147	0.031	0.304	0.086
Observed_features	739.17	745.00	605.17	745.33	21.695	0.042	0.484	0.093
Shannon	7.36^ABa^	7.43^Aa^	6.89^Bb^	7.53^Aa^	0.078	0.008	0.992	0.032
Pielou_e	0.77^ab^	0.78^a^	0.75^b^	0.79^a^	0.005	0.06	0.341	0.145
Dominance	0.01^b^	0.01^b^	0.03^a^	0.01^b^	0.001	0.011	0.507	0.048
Simpson	0.99^a^	0.9^9a^	0.97^b^	0.99^a^	0.002	0.011	0.507	0.048

**Figure 2:**
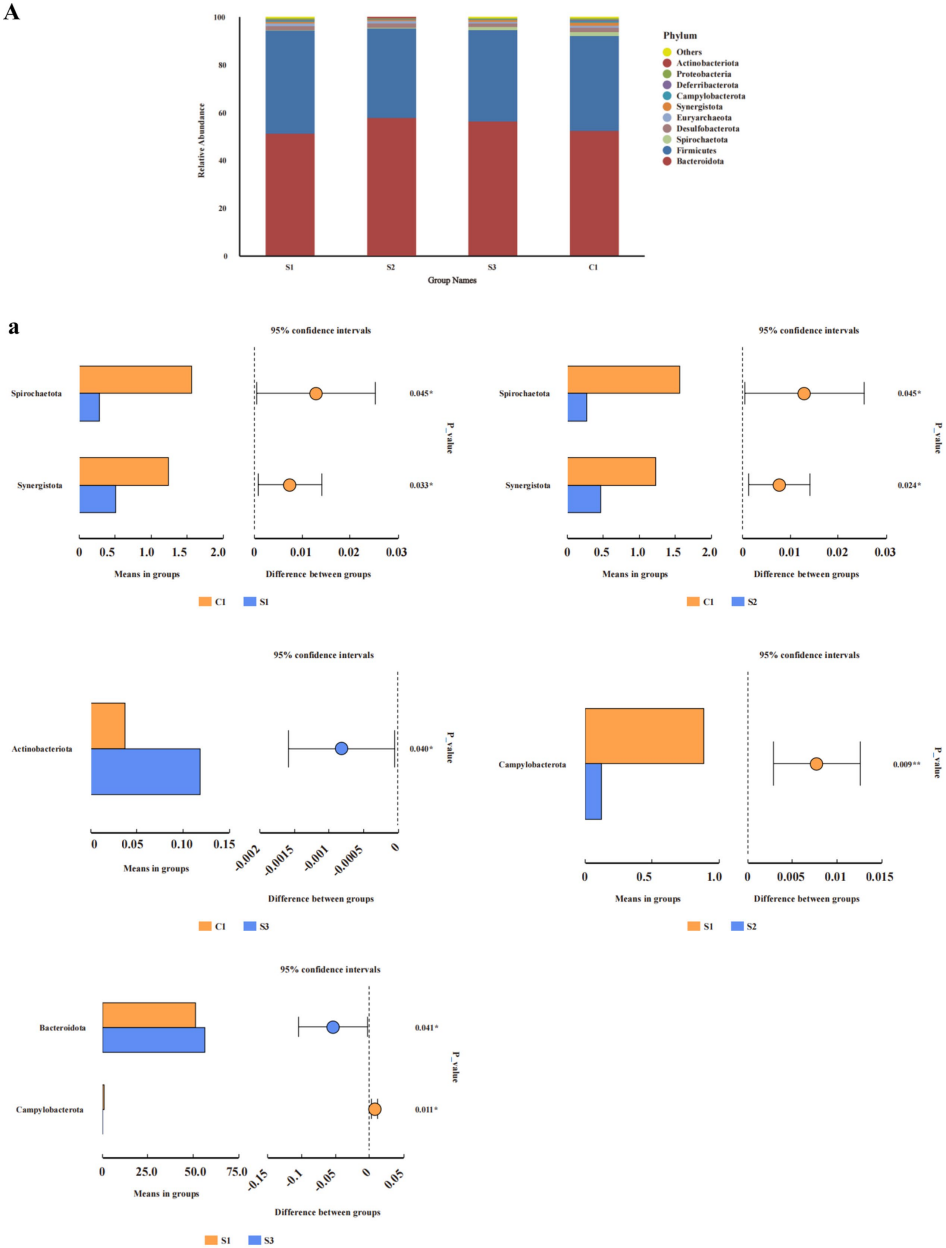
Analysis of relative abundance and compositional differences at the phylum and genus level in cecal contents. Relative abundance of bacteria: **(A)** phylum. Variability of cecal bacteria, **(a)** phylum. S3, 1.5% L-Cit; S2, 1% L-Cit; S1, 0.5% L-Cit; C1, control (CON) group.

From the perspective of phyla (Figure 2A), the relative abundance of Synergistota in each experimental group was significantly lower than that in the control (*p* < 0.05), and the relative abundance of Campylobacterota in the 1.0 and 1.5% groups was significantly lower than that in the 0.5% group (*p* < 0.05). Discriminant analysis of inter-group differences indicated (Figure 2A) that in comparison with the CON group, the relative abundance of Spirochaetota and Synergistota in the 0.5 and 1% groups was significantly lower, while the relative abundance of Actinobacteriota in the 1.5% group was significantly higher. The 1% group had a significantly lower abundance of *Campylobacterota* than the 0.5% group, while the 1.5% group had a significantly higher abundance of Bacteroidia than the 0.5% group (Student’s *t*-test, *p* < 0.05).

**Figure 3:**
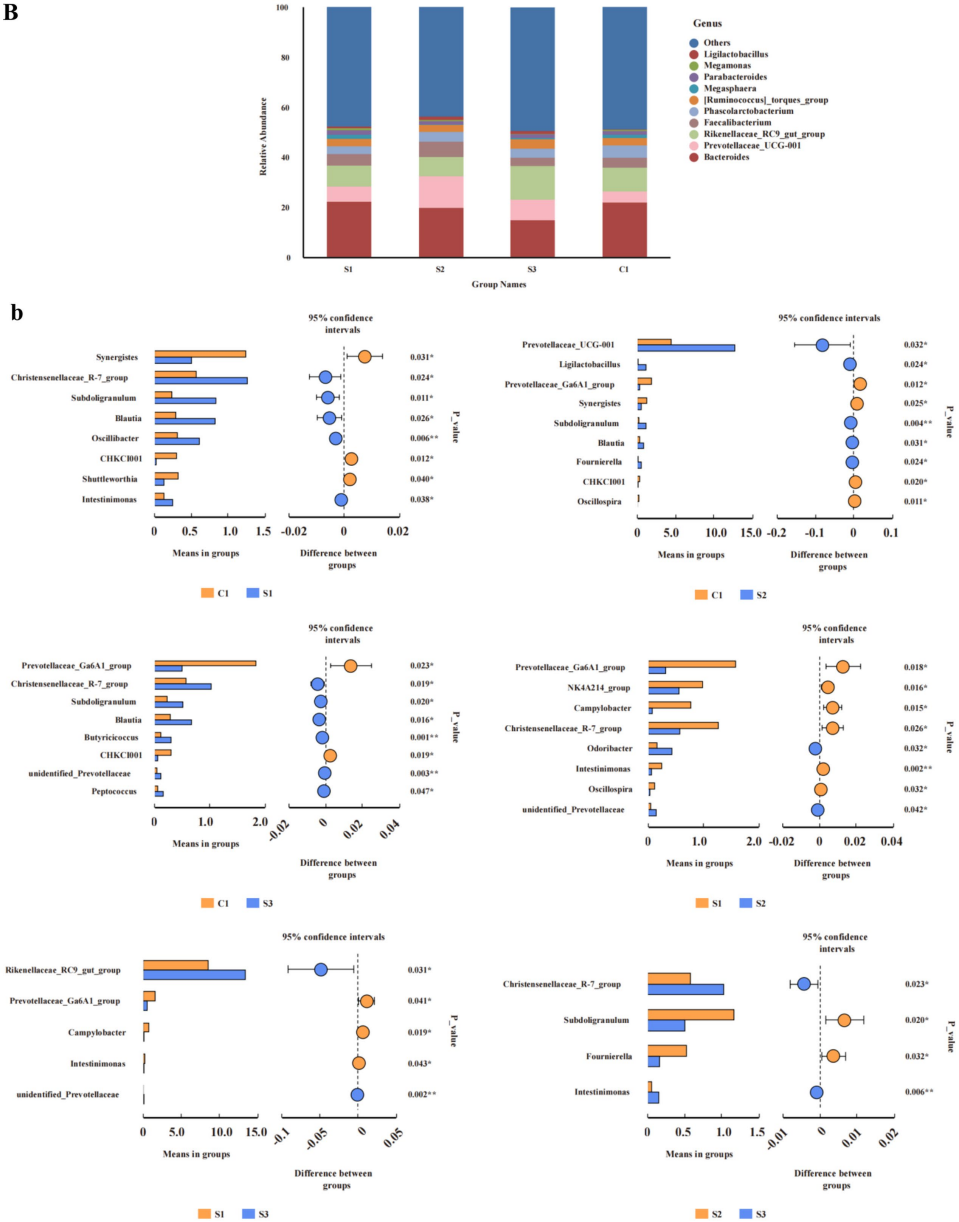
Analysis of relative abundance and compositional differences at the phylum and genus level in cecal contents. Relative abundance of bacteria: **(B)** genus. Variability of cecal bacteria, **(b)** genus. S3, 1.5% L-Cit; S2, 1% L-Cit; S1, 0.5% L-Cit; C1, control (CON) group.

At the genus level (Figure 3B), the relative abundance of Prevotellaceae_UCG-001 in the 1.0% group was significantly higher than in the CON and 0.5% groups (*p* < 0.05). Discriminant analysis of inter-group differences revealed (Figure 3B) that compared to the CON group, the relative abundance of the Christensenellaceae_R-7_group, *Subdoligranulum*, *Blautia*, *Oscillibacter*, and Intestinimonas in the 0.5% group was significantly increased, while the relative abundance of Synergistes and CHKCI001 was significantly decreased; in the 1% group, the relative abundance of Prevotellaceae_UCG-001, *Ligilactobacillus*, *Subdoligranulum*, *Blautia*, and *Fournierella* significantly increased, while the relative abundance of the Prevotellaceae_Ga6A1_group and Synergistes significantly decreased; in the 1.5% group, the relative abundance of the Christensenellaceae_R-7_group, *Subdoligranulum*, *Blautia*, *Butyricicoccus*, unidentified_Prevotellaceae, and *Petococcus* significantly increased, while the relative abundance of the Prevotellaceae_Ga6A1_group and CHKCI001 significantly decreased. Compared with the 0.5% group, the relative abundance of the Prevotellaceae_Ga6A1_group, the NK4A214_group, *Campylobacter*, the Christensenellaceae_R-7_group, and *Intestinimonas* in the 1% group significantly decreased, while the relative abundance of *Odoribacter* significantly increased. In the 1.5% group, the relative abundance of the Prevotellaceae_Ga6A1_group and *Campylobacter* significantly decreased, while the relative abundance of the Rikenellaceae_RC9_gut_group significantly increased (Student’s *t*-test, *p* < 0.05 and *p* < 0.01).

**Figure 4:**
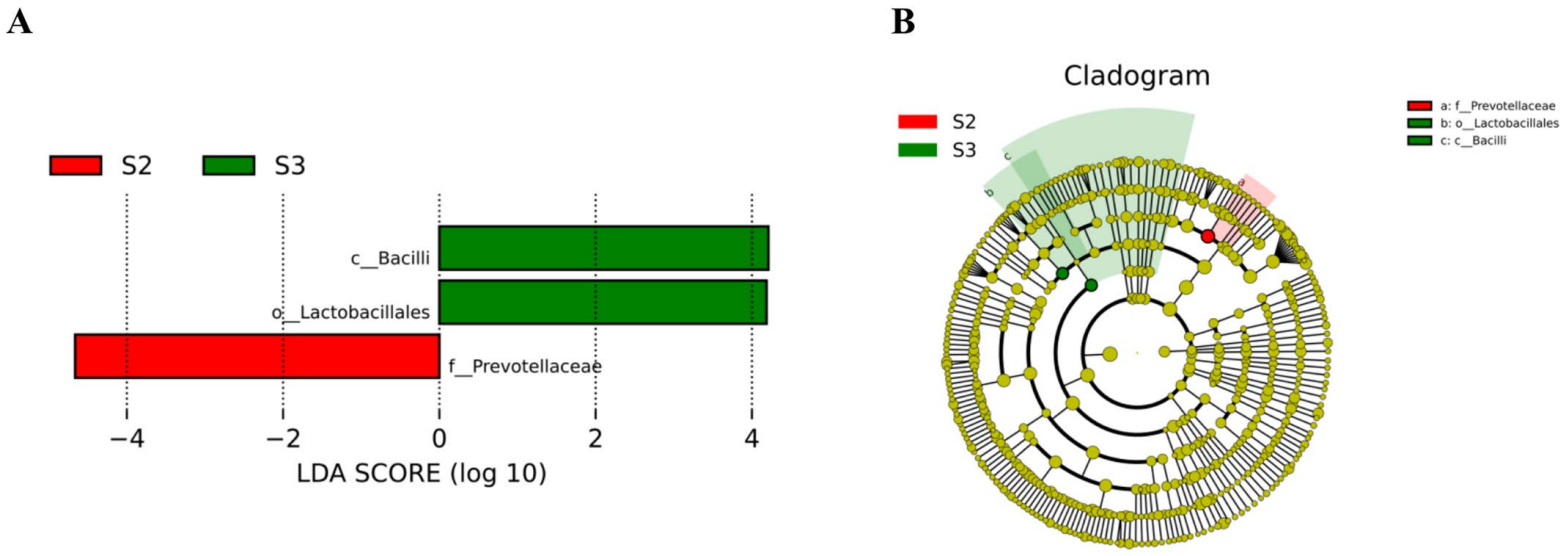
LEfSe analysis. **(A)** LDA fraction of OTUs in cecal microbiota of broilers induced by different concentrations of L-Cit. **(B)** Branch maps showing the phylogenetic distribution of cecal bacteria in yellow-feathered broilers at different L-Cit concentrations. Groups: green, S3, 1.5% L-Cit; red, S2, 1% L-Cit.

The results of LEfSe analysis (Figure 4) show that when LDA = 4, S2 has one potential gut microbiome marker from the family Prevotellaceae, and S3 has two potential gut microbiome markers, including c_*Bacilli*, o_Lactobacillales, and f_Lactobacillaceae.

**Figure 5:**
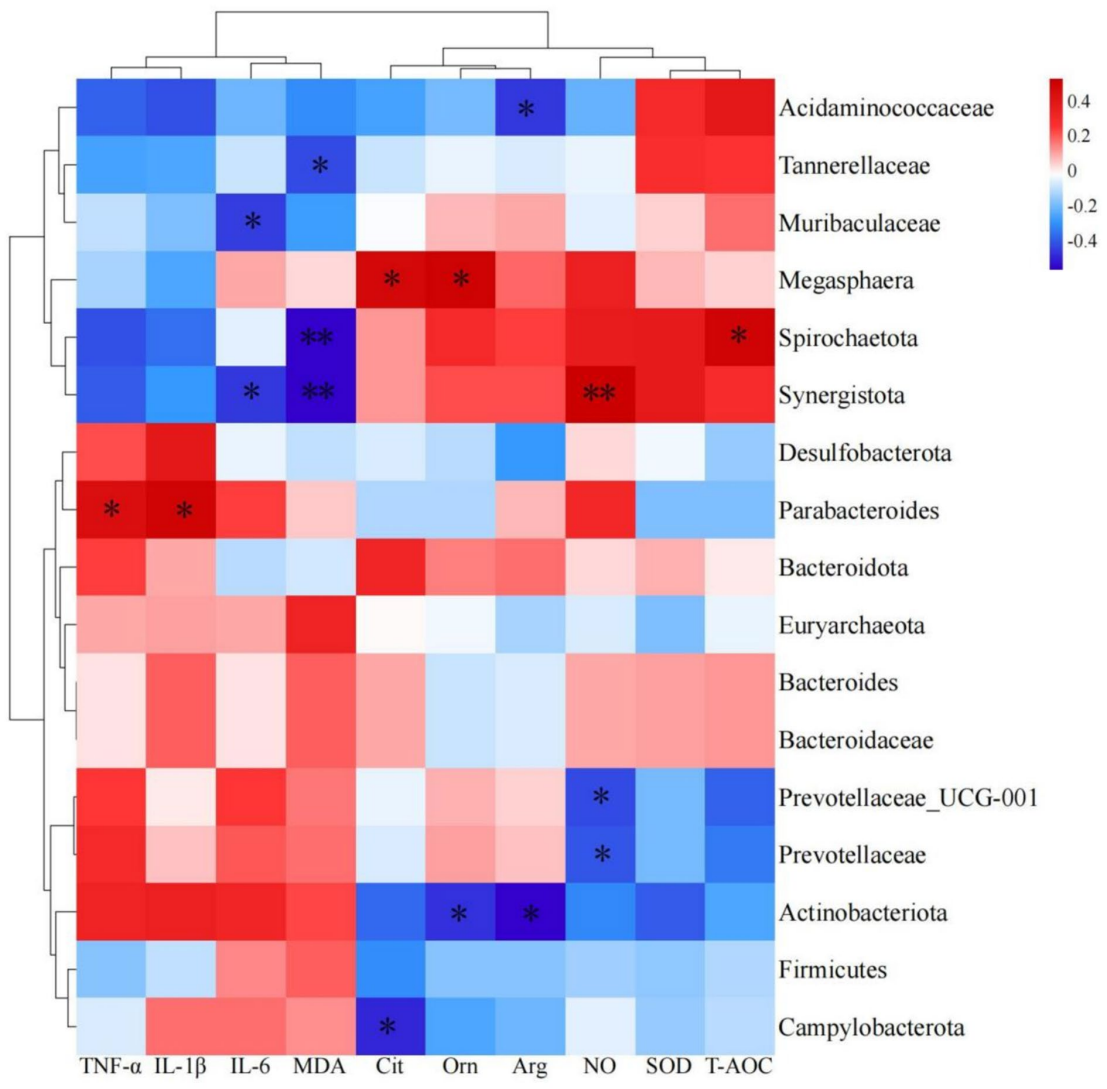
Heatmap of predominant bacteria in cecum with amino acids, related metabolites, antioxidants and immune indexes.

To clarify the interactions between gut microbiota and amino acids, related metabolites, antioxidant, and immune indicators, we conducted a Pearson correlation coefficient analysis (Figure 5). Parabacteroides was significantly correlated, positively, with TNF-*α* and IL-1β (*p* < 0.05); Synergistota exhibited a significantly negative correlation with IL-6 and MDA, and a highly significant positive correlation with NO (*p* < 0.05 or *p* < 0.01); Spirochaetota was significantly correlated, positively, with T-AOC, and significantly negatively correlated with MDA (*p* < 0.01); Muribaculaceae showed a significantly negative correlation with IL-6 (*p* < 0.05); Tannerellaceae had a significantly negative correlation with MDA (*p* < 0.05); Campylobacterota exhibited a significantly negative correlation with Cit (*p* < 0.05); Actinobacteriota showed significantly negative correlations with Orn and Arg (*p* < 0.05); and Prevotellaceae and Prevotellaceae_UCG-001 exhibited significantly negative correlations with NO (*p* < 0.05).

### 3.8 Expression of intestinal compact connectin and anti-inflammatory genes

**Figure 6:**
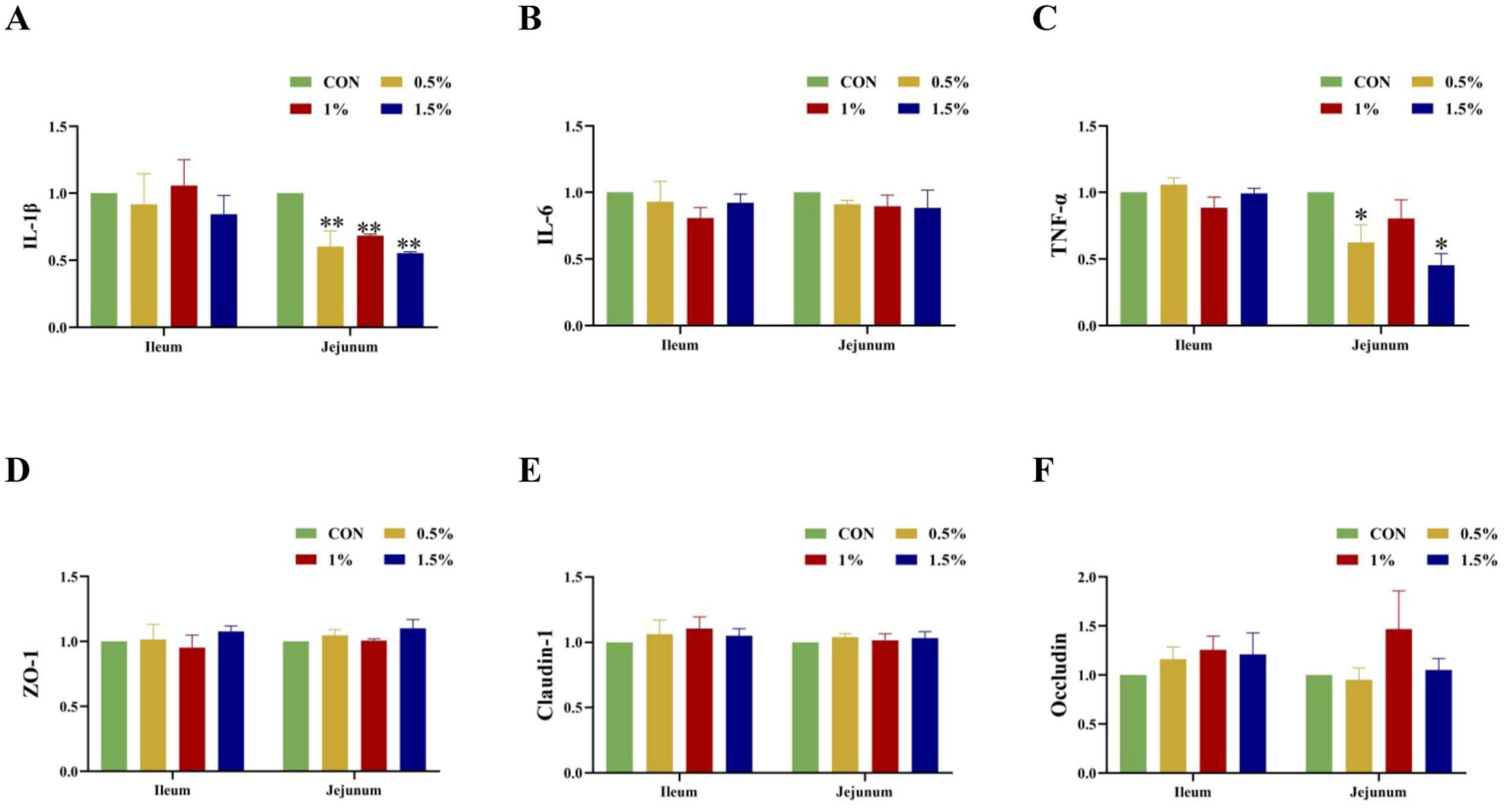
Influence of L-Cit concentration on expression of tight junction proteins and inflammatory factor mRNA in mucosae of the ileum and jejunum (*p* < 0.05 *, *p* < 0.01 **). **(A)** IL-1β, **(B)** IL-6, **(C)** TNF-α, **(D)** ZO-1, **(E)** Claudin-1, **(F)** Occludin mRNA expression in jejunum and ileum.

Figure 5 shows that supplementation with different concentrations of *L*-cit significantly decreased the mRNA expression of IL-1β in jejunal mucosae (*p* < 0.05; Figure 6A), while the 0.5 and 1.5% groups showed significantly decreased TNF-α mRNA expression in jejunal mucosae (*p* < 0.05; Figure 6C).

## 4 Discussion

Adding appropriate amino acids to animal diets can enhance feed intake and improve nutrient utilization. In this study, *L*-Cit supplementation in the diets of yellow-feathered broilers enhanced growth, significantly increasing live weight, carcass weight, eviscerated weight, and thigh muscle weight, in a dose-dependent manner, the improvement in production performance from *L*-Cit supplementation may be related to its direct involvement in the urea cycle. Exogenous citrulline enhances the conversion of ornithine to *L*-1-pyrroline-5-carboxylic acid (P5C) via ornithine transaminase, which subsequently breaks down into glutamate. Both ornithine and glutamate are known to stimulate the release of growth hormone (Chacrabati et al., 2017). On the other hand, the positive growth effect may be related to the fact that citrulline can be converted through the urea cycle to arginine and NO (Baydoun et al., 1994). Being the most abundant nitrogen carrier in tissue proteins, arginine provides a rich nitrogen source and enhances nutrient transport (Hassan et al., 2021), thereby improving growth performance. We also observed that dietary addition of 0.5% or 1% *L*-Cit significantly increased the pH of breast muscle 24 h post-mortem. The pH of muscle affects its water-holding capacity and color, which can reflect the speed and intensity of muscle glycolysis after slaughter. A higher pH value corresponds to a slower rate of glycolysis of muscle glycogen, reduced water exudation, a more stable protein structure, better meat preservation, and a more appealing appearance for consumers. A rapid decrease in pH after slaughter indicates poor muscle water retention, which seriously affects meat tenderness (Zhang et al., 2015). A study examining the addition of 0.5 and 1% *L*-Cit to feed showed that it effectively improved the meat quality of broilers (Maynard et al., 2023) by reducing the shear force of breast muscle, which was consistent with the pH results.

The addition of *L*-Cit to the diet of broilers promoted the Cit- Arg-NO cycle, with significant or extremely significant increases in the concentrations of citrulline, ornithine, and arginine in the 1 and 1.5% groups. Uyanga et al. (2020) found that adding 0.25, 0.5, and 1% L-Cit to the diets of Hyland’s brown laying hens significantly increased the concentration of citrulline, ornithine, and arginine during summer when the average daily minimum and maximum temperatures were 25°C and 31°C, respectively, which is in agreement with this study. This indicates that exogenous supplementation with *L*-Cit can increase the bioavailability of plasma citrulline and arginine. The increase in ornithine may be due to some *L*-Cit entering the liver through the bloodstream (Elkhatib and Buchman, 2012); in the liver, the conversion of *L*-Cit primarily occurs via the urea cycle. The increase in arginine may be the result of reduced consumption from other metabolic pathways, increased biosynthesis of arginine, or increased transport of arginine (Hu et al., 2019).

The concentrations of histidine and tryptophan also significantly increased with the increasing levels of *L*-Cit added. This may be related to the body’s absorption and utilization of the three basic amino acids: arginine, lysine, and histidine in a similar manner. High doses of *L*-Cit may promote the absorption and utilization of other basic amino acids, thereby increasing the concentration of histidine (Neinast et al., 2019). Tryptophan is transported by the sodium-dependent neutral amino acid transporter (ASCT2), which preferentially transports tryptophan and has a high affinity for it (Zeng et al., 2012). *L*-Cit may promote the expression of ASCT2, thereby increasing the concentration of tryptophan. Second, as a non-essential amino acid, *L*-Cit increases the concentration of non-essential amino acids and the total amino acid concentration in plasma, resulting in a significant decrease in the ratio of essential amino acids to non-essential amino acids (EAA/NEAA). This indicates that *L*-Cit can promote the transport of non-essential amino acids, enhance the body’s ability to synthesize proteins, and provide sufficient energy for the body, which could improve growth performance.

Adding *L*-Cit significantly increased the plasma NO and urea levels in broilers but had no significant effect on blood ammonia levels. This result is consistent with the findings of Zhang et al. (2016) and Fligger et al. (1997) which showed that long-term supplementation of arginine increased urea levels in the plasma of pregnant ewes and calves. Exogenous supplementation of *L-*Cit may increase the bioavailability of arginine and NO. Arginine can promote the involvement of carbamoyl phosphate in the urea cycle, thereby increasing urea content (O’Connor et al., 1985). Additionally, it can be seen from the blood biochemical indicators in this study (Table 7) that after adding *L*-Cit to the feed, although the urea content increased, there were no negative effects on the liver and kidneys.

Inflammatory cytokines such as IL-1β, IL-6, and TNF-*α* are key players in the inflammatory response. IL-1β is a typical pro-inflammatory cytokine that induces the release of other inflammatory cytokines such as IL-6 and TNF-α and stimulates T-cell activation, thereby triggering local and systemic inflammatory responses (Zheng et al., 2020) IL-6 attracts and activates inflammatory cells such as neutrophils and monocytes, causing them to migrate to the site of inflammation and release inflammatory mediators (Xing et al., 2021). TNF-α can also stimulate the release of inflammatory mediators from inflammatory cells such as macrophages and neutrophils (Yang S. et al., 2020) that induce the production of IL-1β and IL-6, which enhances the effects of TNF-α. Reducing the levels of these inflammatory cytokines maintains balanced immune activity and enhances the resistance of poultry to pathogens, thus improving growth and feed conversion. In these experiments, adding different concentrations of *L-*Cit significantly enhanced antioxidant and immune activity in broilers, with the specific mechanisms of action as follows: 1. NO pathway: once *L*-Cit enters the body, it is rapidly metabolized into arginine and NO. NO can inhibit the nitrosylation of cysteine residues on the p50 subunit and activate IKB, which binds to NF-κB, thereby inhibiting the nuclear translocation of NF-κB and suppressing the production of pro-inflammatory cytokines and the expression of adhesion molecules (Fathima et al., 2024). 2. Cit- Arg-NO cycle pathway: during the inflammatory response, TNF-α can activate the nuclear transcription factor, NF-κB, which transmits signals to the cell nucleus to initiate transcription of the TNF-α gene and stimulate monocytes and macrophages to produce inflammatory factors such as IL-1, IL-6, and IL-8 (Imbard et al., 2023). NO can inhibit antibody response by regulating the activity of T cells, macrophages, and NK cells (Lan et al., 2020). In this study, we found that tryptophan concentration was significantly increased, and the higher tryptophan levels could alleviate inflammation, which could also be one of the reasons for improving the antioxidant and immune performance of broilers.

The metabolism of arginine by gut bacteria not only affects bacterial growth but also influences their colonization in the small intestine. Conversely, the regulation of amino acid metabolism by arginine in small intestinal bacteria relies on the composition and relative abundance of microbes in the community (Flores et al., 2024). The addition of *L*-Cit significantly increased the relative abundance of Prevotellaceae_UCG-001, while significantly reducing the relative abundance of Synergistota and Campylobacterota, indicating that *L*-Cit can enhance the abundance of dominant bacteria in the cecum, enriching the quantity of gut bacteria in broilers. These increases and decreases in bacterial abundance significantly alter the structure of the cecal microbial community. This suggests that the addition of *L*-Cit to broiler chicken feed may have a positive impact on gut microbial diversity. Amino acids, as nitrogen sources, can interact with gut microbes, and the metabolism and utilization of amino acids by gut microbes promote the growth and development of both the gut microbiota and the host (Table 2). Maslen et al. (2023) found that the reduction in gene content and species abundance of the microbiome is closely related to improved feed efficiency, meaning that lower alpha diversity correlates with higher production performance. In this study, the 1% group showed a significant decrease in alpha diversity and notable increases in production performance and species composition, consistent with the findings of Maslen et al. (2023).

At the same time, differential discriminant analysis showed that the relative abundances of Synergistaceae, Synergistes, Campylobacteraceae, and Campylobacter were decreasing. The reason for this difference may be that the addition of *L*-Cit can increase the total amino acid content in the body. Synergistota can effectively utilize amino acid metabolism to enhance the body’s antioxidant and immune performance, which is consistent with the results of our correlation analysis. The correlation analysis results indicated (Figure 5) that Synergistota was significantly negatively correlated with IL-6 and MDA, while Campylobacterota was negatively correlated with citrulline, suggesting that *L*-Cit exerted anti-inflammatory effects through the Cit-Arg-NO cycle and maintained ecological balance within the intestine. Zampieri et al. (2019) discovered a unique aromatic amino acid transport system in *Escherichia coli*, where high concentrations of amino acid degradation metabolites, such as pyruvate and oxaloacetate, directly inhibited the phosphotransferase system, which may have contributed to the decrease in the relative abundance of Campylobacterota. In this study, the relative abundance of Prevotellaceae_UCG-001, Prevotellaceae_Ga6A1_group, and unidentified Prevotellaceae increased in the 1% *L*-Cit group. Correlation analysis revealed that Prevotellaceae and Prevotellaceae_UCG-001 were significantly negatively correlated with NO, indicating that Prevotellaceae is involved in the metabolism of citrulline. This could result in a synergistic effect between the Prevotellaceae and Synergistota, promoting the anti-inflammatory action of Synergistota through amino acid degradation and reducing the relative abundance of Campylobacterota (Cuevas-Sierra et al., 2020). Parabacteroides is a core member of the gut microbiota, and this symbiotic bacterium has been shown to regulate the host’s mucosal immune system, alleviate inflammation, and participate in carbon metabolism, possessing several polysaccharide utilization sites (Wu, 2023). Correlation analysis in this study showed that Parabacteroides was significantly positively correlated with TNF-*α* and IL-1β, indicating that L-citrulline increases the concentration of arginine in the body, leading to a higher relative abundance of Parabacteroides and a reduction in the secretion of inflammatory factors. Muribaculaceae produces short-chain fatty acids from endogenous mucopolysaccharides and exogenous polysaccharides (dietary fiber) (Zhu et al., 2024). In this study, Muribaculaceae was significantly negatively correlated with IL-6, suggesting that arginine metabolism generates short-chain fatty acids that stimulate the intestinal mucosa and inhibit the NF-κB signaling pathway, thereby exerting anti-inflammatory effects.

To further investigate the differences in gut microbiota composition and species abundance, LEfSe analysis was conducted, revealing significant differences in species such as f_Prevotellaceae, c_Bacilli, and o_Lactobacillales. These findings suggest that the addition of 1% *L*-Cit may reduce the concentration of oxygen in the gut by channeling it through the glycolysis/gluconeogenesis pathway. This reduction could create an anaerobic growth environment favorable for beneficial bacteria such as lactic acid bacteria and bifidobacteria, promoting the proliferation and activation of immune cells in the gut. This process ensures the balance and stability of the gut microecological system, ultimately supporting animal growth (Ilinskaya et al., 2017; Yang L. et al., 2020).

While *L*-Cit has shown positive effects in improving body weight and gut health, excessive intake must be avoided to minimize potential adverse effects on blood glucose, lipids, and gut health. Based on the results of this study, the optimal dietary intake of *L*-Cit in poultry nutrition is 1%. Although *L*-Cit showed positive effects on body weight, antioxidant and immune properties, and intestinal health, further research is needed to investigate its long-term effects on metabolic health.

## 5 Conclusion

Addition of *L*-citrulline can enhance the production performance of broilers and improve antioxidant and immune functions, thereby protecting intestinal health. The recommended amount for feed supplementation is 1 to 1.5%.

## Data Availability

The original contributions presented in the study are publicly available. This data can be found at: https://www.ncbi.nlm.nih.gov/sra/PRJNA1210154.
